# Welfare assessment of horses and mules used in recreational and muleteer work in the Colombian coffee region

**DOI:** 10.3389/fvets.2022.1031192

**Published:** 2022-11-17

**Authors:** Marlyn H. Romero, Fernando Meneses, Jorge A. Sanchez

**Affiliations:** ^1^Department of Animal Health, Faculty of Agrarian and Animal Sciences, University of Caldas, Manizales, Colombia; ^2^Program of Veterinary Medicine, Faculty of Agricultural Sciences, Santa Rosa de Cabal University Corporation—UNISARC, Santa Rosa de Cabal, Colombia

**Keywords:** animal well-being, working equine, developing countries, behavior, health indicators

## Abstract

The welfare of working equids in developing countries is sometimes threatened due to the limited resources and/or knowledge of their owners. The objective of this study is to evaluate the welfare of creole horses and mules using a validated protocol that assesses animal-based indicators. A total of 160 horses and 40 mules from three municipalities in the Colombian coffee-growing region were evaluated by means of direct observation of health and behavioral parameters. A descriptive analysis of the variables expressed in proportions was performed. Interactions between the different measurements were examined using the Chi-squared test. Spearman correlations were used to relate the measurements. Horses and mules demonstrated friendly behavior in front of the evaluators (78.13 and 61.54%, respectively); apathetic or severely depressed behavior was low (10.7 and 17.5%, *P* > 0.05). Significant differences in body condition score (BCS) were observed between mules and horses (*P* < 0.05); eighty percent of the mules and 54.4% of the horses exhibited a healthy body condition score (3 or more on a scale of 1 to 5). Less than 15% of the animals had eye problems, limb deformities, and gait abnormalities. Injuries to the head, withers, spine, ribs/flank, hindquarters, and hind legs were observed in a frequency between 12.5 and 30.43% of the animals, with a higher frequency in horses (*P* < 0.05). Weak correlations (R2 coefficient < 0.5), although statistically significant, were observed between low body conditions and the presence of skin and deeper tissue lesions, systemic health abnormalities, and limb problems (*P* < 0.05). The results indicate that owners care for their animals. However, the presence of skin and deep tissue lesions, especially in horses, suggests that they are subjected to high workloads. Therefore, it is essential to train owners in aspects related to the importance of providing their equids with adequate rest periods to recover from work and develop actions to strengthen human-equine interaction.

## Introduction

In many developing countries around the world, working equids contribute to family livelihoods and perform a wide variety of economic, social, and labor-reducing functions (1, 2), especially in mountainous areas, where motorized vehicles have limited access (3). However, animal welfare can be compromised due to the limited resources and/or knowledge of their owners (4). Several factors affecting the welfare of working equids have been described such as the provision of shelter, adequate feed, appropriate harnesses, veterinary care, provision of medications, and the presence and promotion of programs aimed at improving social awareness of the best animal management practices, among other aspects (2, 5).

Working horses and mules are managed differently from most stabled equids (e.g., leisure horses and horses engaged in competitive events), as they are not kept in stables equipped with special infrastructure. On the contrary, they can sometimes work long hours, pull or transport heavy loads and are often exposed to adverse environmental conditions (4). Therefore, the use of animal-based indicators to assess the welfare state of working equids is recommended because they are considered more reliable and relevant compared to resource-based indicators (6).

In 2011, the United Nations Educational, Scientific, and Cultural Organization (UNESCO) designated the Colombian Coffee Cultural Landscape as a World Heritage Site. The importance of coffee growing in the Colombian Coffee Cultural Landscape has transcended the economic aspect (7). Around this activity, a series of traditions or cultural and social manifestations have developed in the region that that has been transmitted from generation to generation. Among these traditions is the *arrieria*, an activity in which the mule driver, along with his mules and horses, participates. The mules are a species that represent “the strength and endurance of a pack animal capable of traversing the mountainous landscapes” (2). The farmer and coffee producer associations have signed veterinary assistance agreements with public universities in order to monitor the health of these animals. In this context, the objective of this study was to evaluate the welfare of mules and working horses through behavioral and health indicators in the Colombian coffee region, and identify the variables that contribute most to its variation and those that require improvement.

## Materials and methods

### Ethical note

All procedures related to the use and care of the animals strictly followed the Colombian regulation norm, Resolution 001634–2010 as stated by the Colombian Agricultural Institute ICA (8). Permission to conduct the study was approved by the Ethics Committee for Animal Experimentation (Act 24/06/2018, Activities with minimal risk) and the Human Ethics Committee (Act 15/06/2018) at the University of Caldas. Farmers were fully informed about the purpose of the study, and they read/listened and signed an informed consent form and authorization to allow us to use the data collected.

### Characteristics of owners

Information was also obtained on the gender and level of education of the owners.

### Animals and observers

The University of Caldas has had a cooperative partnership for 54 years with the department's coffee growers' cooperatives to carry out biannual medical workshops on a 5-year rotation basis (9 semesters). Previously these workshops were coordinated with the cooperatives' extension promoters, who are responsible for convening the community leaders, who in turn are responsible for disseminating the activities to the coffee growers, who participate voluntarily. In these sessions, veterinary medical consultation, reproductive diagnosis of large species, endoparasite control, administration of multivitamin supplements, small surgeries, promotion and training activities in preventive medicine, husbandry practices, sanitary programs (vaccination, feeding, good livestock practices, parasite control), among others, are carried out. Within the sanitary activities carried out by the veterinary medicine and animal science program of the University of Caldas from March to September 2019, 40 mules and 160 Creole working horses from three municipalities in the department of Caldas were studied: The municipalities being: Riosucio (*n* = 37, 18.4%) (Altitude: 1,729 m, Latitude: 5. 417, Longitude: −75.7 5° 25′ 1″ North, 75° 42′ 0″ West), Manzanares (*n* = 85, 42.3%) (Altitude: 1,933 m, Latitude: 5.25 Longitude: −75. 15 Latitude: 5° 15' 0”' North Longitude: 75° 9' 0”' West) and Pennsylvania (*n* = 79, 39.3%9 (Altitude: 2,165 m, Latitude: 5.383, Longitude: −75.1675° 22′ 59″ North, 75° 10′ 1″ West).

The horses and mules were evaluated by two veterinarians who specialize in animal health and have worked as teachers in the area for more than 15 years. A preliminary pilot test was performed with a group of horses and mules (*n* = 30), belonging to the national police in the city of Manizales, to standardize the evaluation criteria for the behavioral and health variables included in the protocol.

### Assessment of animal welfare indicators

A structured instrument was developed that assessed non-invasive indicators of animal welfare according to the protocol previously standardized by Pritchard et al. (6) and validated by the Brooke Hospital for Animals (“the Brooke”). Welfare indicators included measures of physical health and behavioral responses to human presence and contact. In general terms, and according to the guidelines proposed by Burn et al. (9), the following sequence was followed to take the measures included in the protocol: a) The animal's general alertness was assessed from a distance of at least 3 m for 10 s, before asking for the owner's informed consent, b) Once the informed consent was signed, the observer approached the animal at a normal pace, at a distance of 3 m, looking at the animal's neck or chest. The observer approached at an angle of about 20 degrees (not directly in front of the animal), then stopped 30 cm from the animal's head and recorded its response at the time they stopped, c) The observer walked alongside the animal from its head to its rear and back, keeping a distance of about 30 cm from its body, recording any signs of alertness, d) The observer gently placed their hand under the animal's chin, making just enough contact to support some weight, but not so much as to lift the head. If the animal moved its head away from the hand, the observer did not follow it. This was the first point of physical contact between the observer and the animal unless the animal itself had already initiated contact, d) Indicators related to physical health were then recorded, and finally, gait was assessed when the owner was asked to lead the animal for approximately six steps in a straight line away from the observer and then back toward the observer. The age of each animal was determined by dental chronometry examination by evaluating the incisors. The table with observed behaviors (Table 1) presents a brief description of the indicators evaluated in the animals. Pain behaviors were not explicitly included in the assessment.

**Table 1:** Brief descriptions of the behavioral and physical measures taken as part of a working equine welfare assessment (4, 10).

**Variable**	**Categorizations**	**Brief definition**
**General**		
Age (y)	<5/5–15/>15	Assessed by observing the teeth
Sex	Stallion/gelding/mare/pregnant/mare	N/A
Work type	Arrieria	Agriculture activities, transport of goods by the mountains, transport of people
	Recreative	Transport of people
**Behavior**		
General alertness	Alert	Responding to surroundings, with active movement of the ears toward an existing stimulus. Eyes were usually wide open and head up unless sniffing or eating
	Apathetic or depressed	Passive response to surroundings, ears could be back, or lowered, eyes could be open, half or fully closed, head could be up
Observer approach	Response friendly	Movement of the head toward the observer with relaxed face and the eyes opened but not overly wide, forward turning of the ears
	Aggressive	Attempts to bite, rear, kick or strike with the foreleg
Walk-beside	No response	No obvious response
	Signs of attention	Signs of attention
Chin-contact	Accepts	Shows no response to chin-contact
	Avoids	Moves its head to avoid or reject contact, tense body position including upward holding of the head with tensed muscles and facial expression
**General health**		
Body condition	1–5 (including half-scores)	1, very thin; 5, very fat
Mucous membranes	Normal color	Examination of the gingival, labial, ocular, vaginal and penile mucosa: pink, moist and shiny
	Abnormal	Mucous membranes are pale, congestive, cyanotic or with endotoxemia halo, sticky or dry, and without brightness (dull)
Lesions at commissures of lips	Yes	Lesion of any kind including hair loss, healed lesion, scar
	No	Without lesion
Teeth missing	Yes	At least one tooth missing
	No	All teeth present
Molar hooks or sharp edges	Yes	Present
	No	Absent
Eyes	No abnormalities/abnormal	Healthy eyes
		At least one eye with wet eyelashes, discharge, redness, swelling, opacity, or injury
Coat staring	Yes	Matted
	No	Dry, uneven
Ectoparasites	Present Absent	Ticks, mites, bot eggs, lice, or lice eggs anywhere on the body
Fecal soiling	Present	Fecal soiling on inner thighs or hocks, or diarrhea observed during defecation
	Absent	
Heat stress	Present	Flared nostrils, increased respiratory rate, increased respiratory depth with head movement, apathy
	Absent	
**Skin lesions**		
General lesions	Present	Locations of lesions: breast, and shoulders, ears, forelegs, girth and belly, head, hindlegs, hindquarters, knees, lips, neck, point-of-hock, ribs, flank, tail and tail base, withers, and spine. Superficial/healed, broken skin (skin and immediate subcutaneous layers broken), or deep (visible muscle, tendon or bone)
	Absent (None <4 cm)	
Firing lesions	Yes	Cannon bone, suspensory ligament, flexor tendons, and fetlock joint is visible and distinct from each other in all four legs in one or more legs
	No	
Hoof horn quality	Normal/abnormal	Healthy/abnormalities
Hoof shape	Normal/abnormal	Healthy/abnormalities
Swelling of tendons/joints	Normal	Visual inspection of flexor tendons and fetlock joints; normal or swollen (suspensory ligament, flexor tendons and cannon bones indistinct)
	Abnormal	
Limb deformity	Normal	Lateral or flexural abnormalities of the limbs, excluding cow hocked conformation
	Abnormal	
Cow hocked conformation	Normal	A rotational change of the hindlimb
	Abnormal	
Hoof wall(s) conformation and quality	Normal	Visual inspection of hoof length and height (too long, too short)
	Abnormal	
Sole shape and structure	Normal	Round in horses, Healthy
	Abnormal	Abnormalities: asymmetrical shape, flat/convex or cracked sole, frog narrow, hard or absent, bars absent, or heels contracted
Gait	Normal	Normal
	Abnormal	Any reluctance to put weight on a limb and others (lameness or un-evenness, reluctance to put weight on limb, or uneven head-nodding or hip movement)
Lesions of skin and/or deeper tissues	Normal	Visual inspection of animals with full thickness skin or deeper lesions measuring at least 2 cm × 2 cm or 1 cm × 4 cm (superficial/healed, broken skin (skin and immediate subcutaneous layers broken), or deep (visible muscle, tendon or bone)
	Abnormal	

### Statistical analysis

Software Stata Version 13.0 (College Station, Texas, USA) was used for all the statistical analyses. Animals were considered experimental units. A descriptive analysis was made of the measurements, expressed in proportions of animals that presented the behavioral or health parameter observed in each species evaluated (equines and mules). Following the methodology proposed by Pritchard et al. (6), groups of observations belonging to similar categories were added to form aggregated scores for (a) lack of responsiveness to environmental/handling (general attitude + responsiveness to observer approach + responsiveness to observer walking down the side), (b) low body condition score (mucous membranes + coat condition + diarrhea + skin tent + heat stress), (c) lesions of skin and deeper tissues (firing lesions + swelling lesions + swelling of tendons/joints + deformed limbs + long hoof + hoof too short + sole surface abnormal + hoof horn quality) (6). The interactions between the different measures of behavior and health, as well as the interactions of these measures according to the age of the animals, were examined using a Chi-square test. Spearman rank correlation was then used to relate the measurements. A probability level of *P* < 0.05 was chosen as the limit for statistical significance in all tests, whereas probability levels of *P* < 0.10 and *P* > 0.05 were considered as a tendency.

## Results

### Characteristics of owners

Most owners were male (*n* = 187, 93.0%) and 7% (*n* = 14) were female. 80.5% (*n* = 153) had received primary school education and 19.5% (*n* = 37) had received high school education.

### Characteristics of animals

Table 2 shows a description of the sex, age, and type of work performed by the evaluated animals, showing that the largest proportion of horses (*n* = 160, 80%) and mules were dedicated to Arrieria (muleteering) activity (cultivation of coffee, fruit trees, bananas, and others, as well as the transport of wood, food, work supplies for the farm and transport for their owners) and the rest (*n* = 40, 20%), as recreational horses (companionship and transport for their owners). The animals were distributed in different age groups, with a predominance of animals >5 years, in both horses and mules. The practice of castration of males was frequent (93.33%). Pregnant mares (84.0%) were taken to medical clinics to confirm gestational status by ultrasound examination and are not used for labor in the last third of pregnancy (Table 2).

**Table 2:** Description of work type, sex and age group of 200 equids assessed in three departments of Caldas (Colombia).

	**Specie**					
	**Horses**			**Mule**		
	***n*** = **160 (80%)**			***n*** = **40 (20%)**		
	**Total**	**Work type (%**, ***n*****)**		**Total**	**Work type (%**, ***n*****)**	
		**Arrieria**	**Recreative**		**Arrieria**	**Recreative**
*Sex*						
**Stallion**	9.4 (15)	86.7 (13)	13.3 (2)	0 (0)	0 (0)	0 (0)
**Gelding**	37.3 (60)	93.3 (56)	6.7 (4)	55.0 (22)	95.5 (21)	4.5 (1)
**Mare**	37.3 (60)	80.0 (48)	20.0 (12)	45.0 (18)	94.4 (17)	5.6 (1)
**Pregnant mare**	16.0 (25)	84.0 (21)	12.0 (4)	0 (0)	0 (0)	0 (0)
*Age group (years)*						
**<5**	3.8 (6)	83.3 (5)	16.7 (1)	12.5 (5)	100.0 (5)	0 (0)
**5-15**	19.4 (31)	77.4 (24)	22.6 (7)	22.5 (9)	88.9 (8)	11.1 (1)
**>5**	76.6 (123)	87.9 (109)	12.1 (15)	65.0 (26)	96.1 (25)	3.9 (1)

### Assessment of animal welfare indicators

No significant differences were observed in the behavioral and health indicators according to the type of work performed by the animals (*P* > 0.05). A high proportion of horses and mules presented a response to their environment with a general attitude of alertness (89.31 and 82.5%, respectively), without significant differences (*P* > 0.05). Horses and mules demonstrated friendly behavior in front of the evaluators (78.13 and 61.54%, respectively); apathetic or severely depressed behavior was low (10.7% vs. 17.5%) (*P* > 0.05). Statistically significant differences were found in the indicator of chin contact avoidance between horses and mules, with the negative reaction of the latter being greater (*P* < 0.05).

Eighty percent of the mules and 54.4% of the horses exhibited a healthy body condition score (*P* < 0.05), with a body condition score (BCS) of 3 or more on a scale of 1 to 5 (1, very thin; 5, very fat). The body condition index was positively and statistically significant (*P* < 0.05) and correlated with the presence of coat staring, ectoparasites, the condition of mucous membranes, and skin lesions in the corner of the mouth. Less than 15% of the animals had eye problems, limb deformities, and gait abnormalities. Injuries to the head, withers, spine, ribs/flank, hindquarters, and hind legs were observed in a frequency between 12.5 and 30.43% of the animals, the presence of coat staring and ectoparasites were more frequent in horses than in mules (Table 3). Lesions in the skin and/or deeper tissues showed a high prevalence in horses, particularly in the head, breast/shoulder, withers, spine, and ribs/flanks (*P* < 0.05). Additionally, a long hoof wall, abnormal hoof horn quality, and sole surface abnormal lesions were frequent in both horses and mules. The frequency of hoof abnormalities increased with the increasing age of the animals, and there were statistically significant differences in the frequency of long hoof wall and sole surface abnormal lesions (*P* < 0.05). The prevalence of skin lesions was higher in horses than in mules; animals with an age range between 5 and 15 years presented significant differences in the frequency of skin lesions located in the withers, spine, and ribs/flanks (*P* < 0.05) when compared to younger animals.

**Table 3:** Frequency (%) of behavior and health parameters of working horses and mules (*n* = 200).

	**Species**		*P*-value^*^
**Observations (%)**	**Horses**	**Mule**	
	***n* = 160**	***n* = 40**	
*Behavior*			
**General attitude**			
**Alert**	89.31 (142)	82.5 (33)	0.24
**Apathic/severely depressed**	10.69 (17)	17.5 (7)	
*Responsetoobserverapproach* ^a^			
**No response**	3.13 (5)	7.69 (3)	0.11
**Friendly approach**	78.13 (125)	61.54 (24)	
**Avoidance/aggression**	17.5 (28)	30.77 (12)	
*Walkdownside* ^b^			
**Response**	84.47 (136)	65.0 (26)	0.10
**No response**	15.53 (24)	35.0 (14)	
*Avoids chin contact* ^*c*^			
**Accept**	83.95 (161)	65.0 (26)	***<**0.0 1*
**Avoid**	19.37 (39)	35.0 (14)	
*Body condition score (scales 1-5)*			
**1**	6.88 (11)	0 (0)	***<**0.01*
**2**	38.75 (62)	20.0 (8)	
**3**	42.5 (68)	52.5 (21)	
**4**	11.88 (19)	27.5 (11)	
*Health* ^c^			
Mucous membranes abnormal	10.0 (16)	7.69 (3)	0.12
Lesions at commissures of lips^d^	2.53 (4)	5.0 (2)	0.41
Teeth missing	8.18 (13)	2.56 (1)	*0.06*
Molar hooks or sharp edges	47.80 (76)	44.74 (17)	0.12
Eyes(s) abnormal^e^	3.13 (5)	7.5 (3)	0.20
Coat staring/matted/dry/uneven	27.95 (45)	5.0 (2)	***<**0.01*
Ectoparasites	23.13 (37)	10.0 (4)	*0.06*
Diarrhea under tail	6.29 (10)	0 (0)	0.10
Skin tent (loss of elasticity)	16.77 (27)	7.5 (3)	0.14
Heat stress^f^	1.24 (2)	2.5 (1)	0.55
Firing lesions or scars^d^	62.50 (100)	47.5 (19)	*0.08*
Carpal lesions or scars^d^	11,80 (19)	20.0 (8)	0.17
Hock lesions or scars^d^	11.80 (19)	10.0 (4)	0.75
Swelling of tendons/joints	11.25 (18)	2.5 (1)	*0.09*
Limb deformity^g^	4.38 (7)	5.0 (2)	0.86
Cow hocked conformation	15.63 (25)	12.5 (5)	0.62
Hoof wall(s) too long	43.59 (68)	35.0 (14)	*0.09*
Hoof wall(s) too short	8.23 (13)	12.5 (5)	*0.09*
Hoof horn quality abnormal	48.73 (77)	42.5 (17)	0.48
Sole surface abnormal (RF)	34.18 (54)	31.58 (12)	0.84
Gait abnormal^h^	12.82 (20)	7.69 (3)	0.65
*Lesions of skin and/or deeper tissues* ^i^			
Head	26.09 (42)	12.5 (5)	*0.07*
Ears	12.42 (20)	7.5 (3)	0.38
Neck	6.83 (11)	0 (0)	0.0 9
Breast/shoulder	16.88 (27)	15.0 (6)	0.77
Withers	35.4 (57)	12.5 (5)	***<**0.01*
Spine	30.43 (49)	15.0 (6)	*0.05*
Girth	13.04 (21)	7.5 (3)	*0.09*
Belly	7.45 (12)	0 (0)	*0.07*
Ribs/flank	23.13 (37)	7.5 (3)	*0.03*
Hindquarters	17.5 (28)	7.5 (3)	0.11
Tail/tail base	9.38 (15)	7.5 (3)	0.13
Forelegs (except carpus)	11.8 (19)	2.5 (1)	*0.08*
Hindlegs (except hock)	15.53 (25)	2.5 (1)	*0.03*

a Response to the observer approaching the animal's head from 3 to 5 m away, at an angle of approximately 458 (more acute if the animal is wearing blinkers). Friendly approach: animal turns its head toward the observer. Avoidance/ aggression: animal does one or more of the following: turns head away, moves away, flattens ears, attempts to bite or kick.

b Response to observer walking downside of animal's body at a distance of 30 cm from its side, turning at the tail and walking back to head. Response: any acknowledgment of observer's presence, e.g., ear turn, head turn, move away, kick.

c Proportion of animals with signs of each condition.

d Proportion of animals with lesions of any kind including hair loss, healed lesion, scar.

e Proportion of animals with any abnormality of the eye including ocular discharge.

f Proportion of animals showing most or all of the following: flared nostrils, increased respiratory rate, increased respiratory depth with head movement, apathy.

g Proportion of animals showing lateral or flexural abnormalities of the limbs, excluding cow hocked conformation.

h Proportion of animals showing abnormalities of gait or overt lameness.

i Proportion of animals with full thickness skin or deeper lesions measuring at least 2 cm x 2 cm or 1 cm x 4 cm. Firing, tether, carpus, hock, and lip lesions scored previously were not included.

* Significance of difference in proportion between species by Chi-squared test. Bold values: P < 0.05.

Weak correlations were found, but with statistically significant differences when aggregate behavioral parameters and aggregate health parameters were compared (R2 coefficient<0.5); in contrast, no significant correlation was observed between the behavior called “lack of response to the environment/handling” with low body condition score and skin lesions and deeper tissues (*P* > 0.05), as shown in Table 4.

**Table 4:** Correlations between aggregated behavior and health parameters of working horses and mules (*n* = 200).

**Behavior and health parameters**	**Correlation coefficient**	***P***
Lack of responsiveness to environment/handling^a^		
Low body condition score	−0.07	0.34
Lesions of skin and deeper tissues	−0.10	0.14
Systemic health abnormalities^b^	−0.11	0.02
Limb problems^c^	−0.22	*0.01*
Low body condition score		
Lesions of skin and deeper tissues	0.25	***<**0.01*
Systemic health abnormalities^b^	0.47	***<**0.01*
Limb problems^c^	0.26	***<**0.01*
Lesions of skin and deeper tissues		
Systemic health abnormalities^b^	0.28	***<**0.01*
Limb problems^c^	0.15	*0.03*
Limb problems^c^		
Systemic health abnormalities^b^	0.35	***<**0.001*

a Aggregated score: general attitude + responsiveness to observer approach + responsiveness to observer walking down the side.

b Aggregated score: mucous membranes + coat condition + diarrhea + skin tent + heat stress.

c Aggregated score: firing lesions + swelling of tendons/joints + deformed limbs + hoof too long + hoof too short + sole surface abnormal (RF) + hoof horn quality.

Bold values: P < 0.05.

## Discussion

The evaluated animals may not necessarily represent the welfare status of all working equines in the coffee zone, nor in Colombia, because management conditions may vary between the different geographical areas. Nevertheless, this study was carried out to have a baseline for the welfare status of working equines and mules, to identify causes of suffering, and to establish guidelines for the improvement of the well-being of the animals. Likewise, the implemented protocol is an easy, simple, and economical tool that can be adopted by owners to establish the indicator trends over time and evaluate the impact of the improvements that have been made.

### Characteristics of owners

The owners and handlers of the horses and mules in this study were predominantly men. A similar situation is reported in Romania (3) but differs from that reported by Velázquez-Beltrán et al. (2) in the central region of Mexico, where differences according to gender were not observed. However, the activities in which equines are used were differentiated; women used donkeys to carry water and clothes, while men used a higher proportion of mules and horses for agricultural activities, as described in the coffee region of the present study. Additionally, a greater proportion of animal owners had completed their primary education, as has been described in Mexico (2). This aspect favors the viability of finding work in nearby urban centers in the region evaluated, which also reduces the need to migrate far from their village of origin and to make a living from agricultural activities (2).

### Behavioral indicators

The behavioral observations used in this study have been used to establish an animal's responsiveness to the surrounding environment, and help to identify fear or aggression toward humans (6). Likewise, they allow for inferring human-equid interaction and the implications on the psychological state of the animals (11). Fear is considered a negative affective motivational state and in equine species this is a behavior that represents a serious risk of injury for handlers, resulting in a vicious cycle that increases the severity of restraint and fear (6). In this study, the most predominant behavior in response to the environment was the alertness of the animals. This is considered encouraging because some authors have suggested that general alertness or sensory attention behavior, which includes the reception of visual, auditory, olfactory, and sometimes tactile stimuli, is an important measure of animal welfare, representing an animal's interest or willingness to react positively to any sensory stimulation in the environment (10, 12). However, in this study, 10.7% of the horses and 15.5% of the mules were apathetic. Apathetic animals may require prioritization. Apathetic behavior is considered an indicator of poor animal welfare, possibly associated with problems related to disease, exhaustion, chronic pain, lethargy or depression, dehydration, and inconsistent rough handling, among others. (13). Additionally, chronic low back pain is associated with apathy or lack of sensory responsiveness in horses, according to a study by Rochais et al. (12), who evaluated 100 stable horses observed in their home environment. Therefore, it is important to educate owners in the identification of the causal factors of apathy in their animals, with special emphasis on encouraging consistent handling, humane training based on rewards, and the provision of appropriate food, water, and rest (13, 14).

In this study, horses and mules displayed friendly behavior in front of the evaluators, with horses showing a higher frequency of friendliness. Mules have been described as essential for pack work in difficult mountainous areas and superior to horses and donkeys, due to their better skills, endurance capacity, better hoof quality, lower feed requirements, and greater working longevity (15). However, handlers perceive them as more aggressive and difficult to work with (16, 17). However, mules are creatures of habit and do not react well to changes in their daily routine and to contact with strangers. These animals tend to bond with humans after gaining trust; therefore, these results should be analyzed and interpreted with caution (18). Although the level of empathy of the owners with the horses and mules was not evaluated in this study, the *arrieria* (muleteer) culture transmitted from generation to generation in the Colombian coffee-growing region could have influenced a friendlier response of the animals to contact with two strangers, as there is close contact and a human-equine interaction that has been consolidated over years (2). Likewise, the friendly response to the observers may be related to the levels of empathy that the owners have toward the working equids, as the animals are often considered as family members, thus fostering a closer contact, understanding, and identification of the needs of their animals, as well as the building of routines and strong bonds with their handlers, as has been described in owners of working horses in Chile (19, 20), Brazil (21), and Italy (11). However, other studies conducted in Romania suggest that a particularly emotional relationship between the owner and his/her horse is not usually observed (3); these observed differences between studies may be related to specific geo-cultural factors, individual temperament traits of the animals, breed, interaction practices used by animal owners and handlers, reinforcement of occurrences that trigger positive affective states, and familiarity of the person conducting the test, among other aspects (3, 21).

In the walking alongside test, horses and mules were subjected to another common stimulus (the proximity of humans around them under usual working conditions). However, the frequency of animals with avoidance or fear behavior was higher than that found in the response to the observer approach test but lower than in the chin contact test. It has been suggested that this fear response may be associated with previous negative experiences, which are considered to be stable over time and across situations (9). In this study, the evaluation of the behavioral indicators was performed by two observers unknown to the animals, an aspect that could interfere with the obtained results. Research conducted in Romania evaluated the same behavioral indicators and found that the prevalence of the horses' behavioral responses presented significant differences when the tests were applied by the owner or by an unknown evaluator (3). Other studies with similar tests obtained very different results (1, 10, 15), possibly because of aspects such as: a) the cognitive ability of horses and mules to recognize familiar humans (18, 22), including facial recognition (23), and to remember specific experiences, especially bad ones (3), so it would be logical to obtain different reactions from the animals depending on the familiarity with the person performing the test; b) previous human-equine interactions, which when negative, can lead the animal to have excessive fear reactions, which can limit their use and make them dangerous for the conditions of the handlers (24) in which the test is performed (work routine, strange environment (10); d) individual temperament traits of the animals (3); e) the living environment (resources provided, tasks and demands of the work, climatic conditions, and geo-cultural characteristics, among others) (25); f) genetic characteristics and hybrid vigor, greater cognitive and endurance capacities of mules compared to horses are described (26), g) the experience and training of the observer for the evaluation of behavior and some health indicators such as body condition (27), among other aspects. In future studies, we consider it relevant to perform a comparative evaluation of the behavior of horses and mules in front of an unknown evaluator and the owner to control for possible measurement biases that could have occurred. However, having trained evaluators and a validated protocol were aspects that allowed us to obtain standardized information in this research.

Although no significant differences were found between the prevalence of the responses of horses and mules in three of the behavioral tests evaluated in this study (with the exception of chin contact avoidance), some authors suggest that mules, due to their hybrid nature resulting from the artificial crossbreeding of a mare (*Equus caballus*) and a donkey (*Equus asinus*), have probably acquired innate behaviors characteristic of each parental species; an aspect that is still under study due to a lack of knowledge (25). Mules do not have an evolutionary history in the natural environment as their parents do; therefore, it is even more difficult to infer the effects of domestication on the behavior of these hybrids, especially cognitive abilities and natural behaviors (28). Likewise, mules show more signs of avoidance or fear when an unfamiliar person makes repeated attempts to approach the animal for routine procedures or husbandry tasks (18), an aspect that has also been observed during approach tests conducted by known and unknown persons (6). In Colombia, mules are generally prized animals that are part of the coffee cultural landscape; likewise, Paso Fino mules are used for shows, trail rides, cultural tourism, and for sugarcane crops (29). The coffee culture and the attachment of muleteers toward their horses and mules, which are an important source of livelihood for them and their families, and are considered as family members by their owners (20), could have affected the low prevalence of negative behavioral responses toward unknown evaluators. On the other hand, the owners of the evaluated horses and mules voluntarily attended the veterinary days, which is an indication of their concern, commitment, and positive attitude toward their animals (20, 30). However, further studies that consider behavioral variations between horses and mules are required to develop improvements in the husbandry of these animals, focusing on their own needs and welfare conditions; as well as the awareness by owners of the particular body language and characteristics of mules, because unfortunately, their behavior has been misinterpreted by many in different countries, and harsh equipment, abusive tools, and cruel handling have been used to control them (18).

Body condition scoring in equids is very useful for its ability to detect welfare-relevant conditions, including undernutrition, overnutrition, metabolic disorders, laminitis, suboptimal management, and chronic coping difficulties (27). In this regard, there is a belief that mules are more robust than horses, an aspect that contributes to the lack of adequate care by handlers for their feeding and health needs, which can contribute to malnutrition, inadequate hydration, and, in most cases, overall poor animal welfare conditions (16). However, in this study, horses and mules presented healthy body conditions in a high proportion, this being higher in mules. When analyzing the proportion of adverse health indicators and body injuries due to overwork between horses and mules, the latter presented lower frequencies, therefore, better health conditions and management. These same results were described by Ali et al. (17) in Egypt when comparing the levels of animal welfare between donkeys and mules working in brick kilns, an aspect that revealed a greater adaptation of mules to adverse handling conditions.

### Health problems

Oral diseases are one of the main clinical problems in horses and occupy the third place in the global veterinary diagnosis of this species, with dental abnormalities being responsible for most of the observed conditions, which go unnoticed, and in some occasions may produce pain (31); as is the case of pain caused by the use of headpieces and nosebands, which can press on the dental overgrowths (especially the vertical ridges of the upper teeth 06 and 07), causing trauma to the buccal side of the cheeks and lips, producing pain, biting problems and discomfort in the equine. Injuries induced by the bit or chiffney in the mandibular interdental space (bars of mouth) can occur due to excessive force with the bridle to direct the animals. In most cases, a superficial periostitis or sequestration of the mandibular cortex will occur (32). In the coffee-growing region evaluated, this equipment is not used on *arrieria* (muleteer) horses, only on workhorses that owners use for personal transport. In this study, abnormalities in the wear of premolar and molar teeth were prevalent, an aspect that coincides with previous studies done in Colombia (31, 33). This finding is very important from the point of view of the physical health and fitness for work of the animals, which can even compromise the performance or life of the animal, affect chewing, cause the presence of painful ulcers, periodontal disease, fractures, and loss of dental pieces, and cause deep infections of the alveolus (33). Considering the high prevalence of these problems, the implementation of a routine prophylactic program is recommended in every equine to prevent any form of malocclusion and correct overgrowths or excessive wear in time, and also to initiate treatment based on a correct diagnosis.

Skin lesions were frequently detected on the horses and mules in this study, with greater susceptibility in animals over five years old, an aspect that has also been described in working donkeys in Mexico (34, 35) and Ethiopia (36). Skin lesions (head, withers, spine, and ribs/flank) are associated with saddle and harness quality; these produce severe pain, especially those located in the withers, which can impact an animal's ability to work, particularly when loads are heavy (34, 36). In general, older animals may have a more prominent bone structure, resulting in increased contact that creates injuries from ill-fitting equipment or are the result of cumulative injuries over time. Older animals are more exposed to long working hours and carrying heavy loads during their working lives (35). Additionally, immune defense mechanisms are reduced with advancing age and sometimes their owners pay less attention to the treatment of their wounds (36).

Hoof condition is considered a general indicator of care and management of the animals by their owners. Hoof problems have been described as the most common cause of lameness in horses (27). In this study, the presence of horses and mules with long hoof walls and abnormal hoof horn quality occurred at a high frequency. The use of inadequate shoes and deficiencies in hoof trimming can lead to impaired balance, pressure on different parts of the hoof, stress on ligaments and/or tendons, and, finally, permanent gait disturbance (37). Lack of hoof care in animals may be related to poor management by the animal's owner and insufficient training strategies. Factors associated with poor owner management of their animals include economic constraints, lack of knowledge about management practices, the quality of human-horse interaction, owner attitudes, and insufficient owner commitment, among others (38, 39). Studies in India (37) and the United Kingdom demonstrated that long-term (2-year) participatory intervention projects involving animal owners, professionals, and handlers were successful in reducing limb problems and lameness in working horses, promoting adherence to treatment/care plans and positively impacting the quality of human-horse interaction. This style of intervention avoids confrontation and supports clients through a joint exploration of their beliefs, attitudes, and goals as a basis for supporting change in behavior (40).

### Health problems and association with behavioral indicators

A low body condition in horses and mules was correlated with a lack of responsiveness to environment/handling, skin and deeper tissues lesions, systemic health abnormalities, and limb problems suggesting that working equids in poor health show an unresponsive behavioral profile, consistent with sickness, exhaustion, chronic pain, or depression-like states (9, 11). Likewise, it appears that equids with more severe physical problems enter a state of behavioral unresponsiveness, as is the case with animals in low body condition. The causes of low body condition are multifactorial and are likely to include malnutrition, overwork, parasitism, and disease, which could simultaneously cause behavioral unresponsiveness. Furthermore, apathy can lead to reduced appetite, as in sickness and depression, which in turn lowers body condition, as described by Burn et al. (10).

Added behavioral indicators in horses and mules in the coffee-growing area studied were correlated with the presence of systemic health abnormalities and limb problems. Studies in Afghanistan, Egypt, Ethiopia, Guatemala, India, Jordan, Kenya, Pakistan, and the Gambia suggest that equids with more severe physical problems enter a state of behavioral unresponsiveness because the animals' resources are being stretched to their limits and their fitness is compromised; likewise, as a “prey species”, equids conserve their “energy” reserves as a survival strategy, even at the risk of not responding adequately to potentially threatening stimuli (9). This lack of response has been associated with different states of negative well-being, such as overwork exhaustion, chronic pain, apathy or depression, and general malaise (41, 42).

## Conclusions

The low proportion of health and behavioral problems found in the study suggests that owners are concerned about the welfare of their working animals; however, it is important to emphasize that animals whose owners are not concerned about their medical care can be at risk of deteriorating health. The coffee culture and the attachment of the *arrieros* (muleteers) toward their horses and mules, which are an important source of livelihood for them and their families, could be factors that influenced these results. Therefore, independently of the level of schooling and economic possibilities of the owners, the results suggest that a good standard of working animal welfare can be achieved, because cultural factors and the desire and willingness to care for their animals are essential factors in favoring welfare. However, the presence of skin and deep tissue lesions, especially in horses, suggests that they are subjected to high workloads. Therefore, it is essential to train owners in aspects related to the importance of providing their equids with adequate rest periods to recover from work, and promote working hours that are in keeping with their health conditions. Collaborative interventions involving academia, animal owners and handlers could be the way forward for the shared exploration of knowledge, beliefs, attitudes and goals, as a basis for supporting behavioral change and positive human-equine interaction.

## Data availability statement

The raw data supporting the conclusions of this article will be made available by the authors, without undue reservation.

## Ethics statement

The animal study was reviewed and approved by Ethics Committee for Animal Experimentation (Act 24/06/2018, Activities with minimal risk) and the Human Ethics Committee (Act 15/06/2018) at the University of Caldas.

## Author contributions

MR assisted with the conception, design of the experiment, preparation, data analysis, and preparation of manuscript. FM prepared the data for analysis and analyzed the data under the guidance of MR and JS. MR, JS, and FM contributed to interpretation of the results. MR and JS drafted and edited the manuscript. All authors read and approved the final manuscript.

## Funding

This work was based on research supported by Research Investigation and the vice-chancellor of the University of Caldas.

## Conflict of interest

The authors declare that the research was conducted in the absence of any commercial or financial relationships that could be construed as a potential conflict of interest.

## Publisher's note

All claims expressed in this article are solely those of the authors and do not necessarily represent those of their affiliated organizations, or those of the publisher, the editors and the reviewers. Any product that may be evaluated in this article, or claim that may be made by its manufacturer, is not guaranteed or endorsed by the publisher.

## References

[1] Pritchard JC. Animal traction and transport in the 21st century: getting the priorities right. Vet J. (2010) 186:271–4. 10.1016/j.tvjl.2010.08.004 20833088

[2] Velázquez-Beltrán LG Sánchez-Vera E Nava-Bernal EG Arriaga-Jordán CM. The role of working equines to livelihoods in current day campesino hill-slope communities in central Mexico. Trop Anim Health Prod. (2011) 43:1623–32. 10.1007/s11250-011-9881-6 21637993

[3] Popescu S Diugan E-A. The relationship between behavioral and other welfare indicators of working horses. J Equine Vet Sci. (2013) 33:1–12. 10.1016/j.jevs.2012.04.001

[4] Ali ABA El Sayed MA Matoock MY Fouad MA Heleski CR. A welfare assessment scoring system for working equids—A method for identifying at risk populations and for monitoring progress of welfare enhancement strategies (trialed in Egypt). Appl Anim Behav Sci. (2016) 176:52–62. 10.1016/j.applanim.2015.12.001

[5] Mekuria S Mulachew M Abebe R. Management practices and welfare problems encountered on working equids in Hawassa town, Southern Ethiopia. J Vet Med Anim Heal. (2013) 5:243–50. 10.5897/JVMAH10.017

[6] Pritchard JC Lindberg AC Main DCJ Whay HR. Assessment of the welfare of working horses, mules and donkeys, using health and behaviour parameters. Prev Vet Med. (2005) 69:265–83. 10.1016/j.prevetmed.2005.02.002 15907574

[7] Mayorga CDA. Paisaje Cultural Cafetero, Patrimonio de la Humanidad. La cuestión del discurso patrimonial en contraste con el paisaje de la caficultura. Territorios. (2015) 32:35–59. 10.12804/territ32.2015.02

[8] Instituto Colombiano Agropecuario I. Reglamento que establece los requisitos para la expedición de licencias zoosanitarias de funcionamiento que autorizan las concentraciones de animiales y se señalan los requisitos sanitarios para los animales que participen en ellas. D Of la República, Resolución 001634. (2010). Available online at: https://www.ica.gov.co/getdoc/016f3c96-a458-4fa6-ae96-41d18b2221f5/requisitos-sanitarios-y-de-inocuidad-en-la-producc.aspx (accessed November 2, 2019).

[9] Burn CC Dennison TL Whay HR. Environmental and demographic risk factors for poor welfare in working horses, donkeys and mules in developing countries. Vet J. (2010) 186:385–92. 10.1016/j.tvjl.2009.09.016 19926316

[10] Burn CC Dennison TL Whay HR. Relationships between behaviour and health in working horses, donkeys, and mules in developing countries. Appl Anim Behav Sci. (2010) 126:109–18. 10.1016/j.applanim.2010.06.007

[11] Dalla Costa E Dai F Murray LAM Guazzetti S Canali E Minero M. study on validity and reliability of on-farm tests to measure human–animal relationship in horses and donkeys. Appl Anim Behav Sci. (2015) 163:110–21. 10.1016/j.applanim.2014.12.007

[12] Rochais C Fureix C Lesimple C Hausberger M. Lower attention to daily environment: a novel cue for detecting chronic horses' back pain? Sci Rep. (2016) 6:20117. 10.1038/srep20117 26823123 PMC4731760

[13] Hall C Goodwin D Heleski C Randle H Waran N. Is there evidence of learned helplessness in horses? J Appl Anim Welf Sci. (2008) 11:249–66. 10.1080/10888700802101130 18569222

[14] Kumar N Fisseha KK Shishay N Hagos Y. Welfare assessment of working donkeys in Mekelle city, Ethiopia. Glob Vet. (2014) 12:314–9. 10.5829/idosi.gv.2014.12.03.82120

[15] Lagos J Rojas M Rodrigues JB Tadich T. Perceptions and attitudes towards mules in a group of soldiers. Animals. (2021) 11:1009. 10.3390/ani11041009 33916720 PMC8067085

[16] Ali ABA Matoock MY Fouad MA Heleski CR. Are mules or donkeys better adapted for Egyptian brick kiln work? (Until we can change the kilns) *J Vet Behav*. (2015) 10:158–65. 10.1016/j.jveb.2014.12.003

[17] Ali ABA El Sayed MA McLean AK Heleski CR. Aggression in working mules and subsequent aggressive treatment by their handlers in Egyptian brick kilns—Cause or effect? J Vet Behav. (2019) 29:95–101. 10.1016/j.jveb.2018.05.008

[18] McLean AK Navas González FJ Canisso IF. Donkey and mule behavior. Vet Clin North Am Equine Pract. (2019) 35:575–88. 10.1016/j.cveq.2019.08.010 31672203

[19] Luna D Vásquez R Rojas M Tadich T. Welfare status of working horses and owners′ perceptions of their animals. Animals. (2017) 7:56. 10.3390/ani7080056 28788109 PMC5575568

[20] Luna D Vásquez R Yáñez J. Tadich^*^ T. The relationship between working horse welfare state and their owners' empathy level and perception of equine pain. Anim Welf. (2018) 27:115–23. 10.7120/09627286.27.2.115

[21] Hötzel MJ Vieira MC Leme DP. Exploring horse owners' and caretakers' perceptions of emotions and associated behaviors in horses. J Vet Behav. (2019) 29:18–24. 10.1016/j.jveb.2018.10.002

[22] Sankey C Henry S André N Richard-Yris M-A Hausberger M. Do horses have a concept of person? PLoS ONE. (2011) 6:e18331. 10.1371/journal.pone.0018331 21479184 PMC3068175

[23] Stone SM. Human facial discrimination in horses: can they tell us apart? Anim Cogn. (2010) 13:51–61. 10.1007/s10071-009-0244-x 19533185

[24] Waiblinger S Boivin X Pedersen V Tosi M-V Janczak AM Visser EK. Assessing the human–animal relationship in farmed species: a critical review. Appl Anim Behav Sci. (2006) 101:185–242. 10.1016/j.applanim.2006.02.001

[25] Luz MPF Maia CM Gonçalvez HC Puoli Filho JNP. Influence of workload and weather conditions on rolling behaviour of horses and mules. Behav Processes. (2021) 189:104433. 10.1016/j.beproc.2021.104433 34090953

[26] Proops L Burden F Osthaus B. Mule cognition: a case of hybrid vigour? Anim Cogn. (2009) 12:75–84. 10.1007/s10071-008-0172-1 18636282

[27] Hemsworth LM Jongman E Coleman GJ. Recreational horse welfare: The relationships between recreational horse owner attributes and recreational horse welfare. Appl Anim Behav Sci. (2015) 165:1–16. 10.1016/j.applanim.2014.11.01933499202

[28] Burden F Thiemann A. Donkeys are different. J Equine Vet Sci. (2015) 35:376–82. 10.1016/j.jevs.2015.03.005

[29] Mclean A. Comparing the physiological and biochemical parameters of mules and hinnies to horses and donkeys Amy K. McLean, PhD North Carolina State University Proceedings in the Donkey and Mule Welfare Symposuim, Hydra, Greece October (2015).

[30] Haddy E Brown J Burden F Raw Z Kaminski J. Proops L. “What can we do to actually reach all these animals?” Evaluating approaches to improving working equid welfare. PLoS ONE. (2022) 17:e0273972. 10.1371/journal.pone.0273972 36084063 PMC9462723

[31] Cruz Amaya JM Sánchez VJA Vera Hernández LG. Caracterización y prevalencia de las enfermedades orales en el caballo criollo, departamento de Caldas, Colombia. Rev Med Vet (Bogota). (2012) 39:73. 10.19052/mv.73

[32] Dixon PM Dacre I. review of equine dental disorders. Vet J. (2005) 169:165–87. 10.1016/j.tvjl.2004.03.022 15727909

[33] Buitrago-Mejía J Díaz-Cueto M Suarez-Chica A Cardona-Álvarez J. Distribución geográfica de la casuística clínica equina del servicio ambulatorio de grandes animales de la universidad de córdoba, colombia. Rev Científica. (2017) XXVII:270–81.

[34] Burden FA Du Toit N Hernandez-Gil M Prado-Ortiz O Trawford AF. Selected health and management issues facing working donkeys presented for veterinary treatment in rural Mexico: some possible risk factors and potential intervention strategies. Trop Anim Health Prod. (2010) 42:597–605. 10.1007/s11250-009-9462-0 19784862

[35] Galindo F de Aluja A Cagigas R Huerta LA Tadich TA. Application of the hands-on donkey tool for assessing the welfare of working equids at Tuliman, Mexico. J Appl Anim Welf Sci. (2018) 21:93–100. 10.1080/10888705.2017.1351365 28762781

[36] Fsahaye S Kumar N Kebede E Abebe N. Health and welfare assessment of working donkeys in and around Rama town, Tigray, Ethiopia. Ethiop Vet J. (2018) 22:26. 10.4314/evj.v22i1.3

[37] Reix CE Dikshit AK Hockenhull J Parker RMA Banerjee A Burn CC. Two-year participatory intervention project with owners to reduce lameness and limb abnormalities in working horses in Jaipur, India. PLoS ONE. (2015) 10:e0124342. 10.1371/journal.pone.0124342 25898014 PMC4405470

[38] Pearson NY,. A Study of Horse Ownership Management in Victoria. Australia. (2003). Available online at: http://hdl.handle.net/11343/114868 (accessed September 20, 2022).

[39] Paul H Hemsworth GJC. Human-livestock interactions: The stockperson and the productivity and welfare of intensively farmed animals. 2nd ed. Wallingford UK: CABI International. (2011).

[40] Lynden J Hollands T Ogden J A. Farrier making every contact count: a microlevel analysis of farrier-client interaction for partnership working in managing a horse with laminitis. J Equine Vet Sci. (2020) 87:102924. 10.1016/j.jevs.2020.102924 32172914

[41] Ashley FH Waterman-Pearson AE Whay HR. Behavioural assessment of pain in horses and donkeys: application to clinical practice and future studies. Equine Vet J. (2010) 37:565–75. 10.2746/042516405775314826 16295937

[42] Pritchard JC Burn CC Barr ARS Whay HR. Haematological and serum biochemical reference values for apparently healthy working horses in Pakistan. Res Vet Sci. (2009) 87:389–95. 10.1016/j.rvsc.2009.05.003 19552930

